# Comparison of Archaeal Communities in Mineral Soils at a Boreal Forest in Finland and a Cold-Temperate Forest in Japan

**DOI:** 10.1264/jsme2.ME17100

**Published:** 2017-11-07

**Authors:** Reika Isoda, Shintaro Hara, Teemu Tahvanainen, Yasuyuki Hashidoko

**Affiliations:** 1 Research Faculty of Agriculture, Hokkaido University Sapporo, Hokkaido Japan; 2 Department of Biology, University of Eastern Finland Joensuu Finland

**Keywords:** soil archaea, *Thaumarchaeota*, *Thermoplasmata*, volcanic lapilli soil, *amoA* gene

## Abstract

Archaeal communities in mineral soils were compared between a boreal forest in Finland and cold-temperate forest in Japan using 16S rRNA gene-targeted high-throughput sequencing. In boreal soils, *Thaumarchaeota* Group 1.1c archaea predominated and *Thaumarchaeota* Group 1.1a-associated and Group 1.1b archaea were also detected. In temperate soils, *Thaumarchaeota* Group 1.1a-associated and Group 1.1b archaea were dominant members at the subsurface, whereas their dominancy was replaced by *Thermoplasmata* archaea at the subsoil. An analysis of the ammonia monooxygenase subunit A gene of *Archaea* also indicated the distribution of *Thaumarchaeota* Group 1.1a-associated and Group 1.1b archaea in these soils.

*Archaea* are now widely accepted to play important roles in the carbon and nitrogen cycles in various ecosystems (6, 8, 14). A large part of the mesophilic *amoA*-harboring *Archaea* of the phylum *Thaumarchaeota* found in subsoils adapted ammonia oxidation under acidic conditions or low ammonia availability (2, 11). A larger number of the archaeal *amoA* copy has been detected than bacterial *amoA* genes (12), and ammonium-oxidizing *Archaea* have been found in several soil environments, including forests and agricultural lands (5). Some strains within *Thaumarchaeota* Group 1.1a, Group 1.1a-associated, and Group 1.1b were directly shown to be ammonia oxidizers (8, 11, 16, 17), whereas evidence for ammonia oxidization by members in Group 1.1c has not yet been obtained (10). The abundance of *Thaumarchaeota* Group 1.1c and *Thermoplasmata*, which are generally found in hot environments, was found to increase with the depth of temperate acidic soil, which is a loamy sand with a pH of 3.8–4.3, in Germany (7). Furthermore, pyrosequencing-based investigations demonstrated that *Archaea*, particularly *Thaumarchaeota*, are abundant significantly in a mineral horizon (10–20 cm) than an organic horizon (0–10 cm) in a planted Norway spruce forest in France (18). However, limited information is currently available on archaeal communities in acidic forest soils inside the Arctic Circle in Finland. Weedon *et al.* previously reported that, in contrast to the fluctuations observed in bacteria, those in soil mesophilic ammonia-oxidizing *Archaea* (AOA) were rarely affected by temperature increases, and this may be related to the levels of water-soluble organic carbon (WSOC) (20). However, the relationship between archaeal community structures and WSOC concentrations has not yet been elucidated in detail. Therefore, we herein investigated archaeal community structures in mineral soils from two different types of forest beds: the mineral horizons of a mixed conifer forest edge in Finland inside the Arctic Circle, in which the litter supply and its decomposition are limited and slow, and a planted larch forest in northern Japan, in which a large amount of litter is supplied and rapidly decomposed. In the present study, we focus on archaeal communities in the soils of mineral horizons collected from boreal and temperate forests in order to provide insights into archaeal community distributions among different forest sites (Fig. 1).

The subsoil of the boreal forest was collected from Pallas National Park, Finland, which is located inside the Arctic Circle (68°02′N, 24°04′E, altitude 514 m). B1- and B2-horizon soils were collected. Their pH values were 5.12 and 5.20, respectively, while WSOC were 10.3 and 10.6 mg kg^−1^, respectively. The subsoil of a cold-temperate forest was collected from Tsutamori Forest Park in Tomakomai, Japan (42°39′N, 141°47′E, altitude 25 m), which is a planted larch forest (>40 years old) established on a volcanic sand/lapilli bed. A- and B-horizon soils were collected. Their pH values were 5.64 and 6.58, respectively, while WSOC were 43.4 and 16.9 mg kg^−1^, respectively. Full methods for pH and WSOC measurements are described in Supplementary Information.

The archaeal communities in the Pallas B1- and B2-horizons of soils and Tomakomai A- and B-horizons of soils, which were analyzed using a high-throughput short-read sequencer, were dominated by the phyla *Thaumarchaeota* and *Euryarchaeota* (Fig. 2). The major thaumarchaeal reads, except for those of the marine group (MG), were observed, while most of the euryarchaeal reads were identified as those of class *Thermoplasmata* (3, 4). *Thaumarchaeota* Group 1.1, previously known as “Finnish Forest Soil *Archaea*”, is widely distributed under vegetative acidic soil at pH 5 or less (1, 10), and is one of the most abundant groups of soil *Archaea* in the B1- and B2-horizon soils of Pallas. *Thaumarchaeota* Group 1.1c is adapted to soils with a relatively low pH and high water content, and some subgroup members also prefer high organic matter and low nitrate concentrations in soils (15). *Thaumarchaeota* Group 1.1a-associated, including the obligate acidophilic AOA ‘*Candidatus* Nitrosotalea devanaterra’ (11), dominated in the mineral horizons of soils from the planted larch forest in the Tomakomai site. The A-horizon soil of the site was very rich in Group 1.1a-associated archaea (approximately 50% of the archaeal population in A-horizon soil). In addition, the A-horizon soil of the Tomakomai site was uniquely abundant in another thaumarchaeal group of the *Nitrososphaera* cluster (Group 1.1b), including the soil AOA *Nitrososphaera viennensis* (16, 17) (Fig. 2).

Members of the class *Thermoplasmata* were also the abundant archaeal class in these mineral horizon soils. The B1-and B2-horizon soils from Pallas were uniquely rich in *Thermoplasmata* Thp-A-102 (sediment group), which was initially detected in geothermal spring water in Greece at a depth of 20–30 cm below the surface (9). The population ratios of *Thermoplasmata* Thp-A-102 in the B1- and B2-horizon soils were 42 and 36%, respectively. In contrast, more than 70% of the sequence from the B-horizon soil of Tomakomai was identified as the *Thermoplasmata* group, mainly composed of terrestrial and fresh water groups (50 and 26%, respectively), while A-horizon soil contained uniquely unclassified *Thermoplasmata*. Thus, marked differences were observed in the community structures of the class *Thaumarchaeota* between these two sites (Fig. 2).

Along with a 16S rRNA gene-based high-throughput analysis for archaeal community structures, the ammonia monooxygenase subunit A (*amoA*) gene of *Archaea* from soils was cloned after the separation of *amoA* gene-targeted PCR-denaturing gradient gel electrophoresis (DGGE) bands. A phylogenetic analysis indicated that two groups of *amoA* genes derived from *Thaumarchaeota* Group 1.1a-associated and Group 1.1b co-existed in boreal forest soils and the A-horizon soil of the temperate forest (Fig. 3a, b).

Hernández *et al.* suggested that ammonia oxidizers in newly emergent volcanic sand/lapillus soil are pioneers that promote the development of carbon and nitrogen sinks necessary for biodiversity enrichment in the soil ecosystem (3). Hence, *Thaumarchaeota* Group 1.1a-associated and Group 1.1b dominant in A-horizon soil at the Tomakomai site may be one of them. This is the first study on the archaeal community structure of volcanic sand/lapillus soil under a planted larch forest in Japan. The composition of *amoA* genes may serve as a clear indicator of the unique dynamics of the thaumarchaeal AOA at different sites and depths of mineral soils, reflecting overall local soil conditions and the nitrification rate. Conversely, *Thaumarchaeota* Group 1.1c in boreal forest soil has already been reported to neither harbor any functional *amoA* gene nor show evidence of ammonia oxidation (19). Thus, the ecological function of Group 1.1c *Thaumarchaeota* is poorly understood; however, the abundance of Group 1.1c and the mesophilic groups of euryarchaeal *Thermoplasmata* may contribute to C mineralization (13).

Although our results highlight only limited members of the microbial community at a few sites (or were even biased due to the soil preservation method stocked at 4°C), the ecological roles of *Thaumarchaeota* Group 1.1c and other soil *Archaea* warrant further study as an important research target.

## Supplemental information



## Figures and Tables

**Fig. 1:**
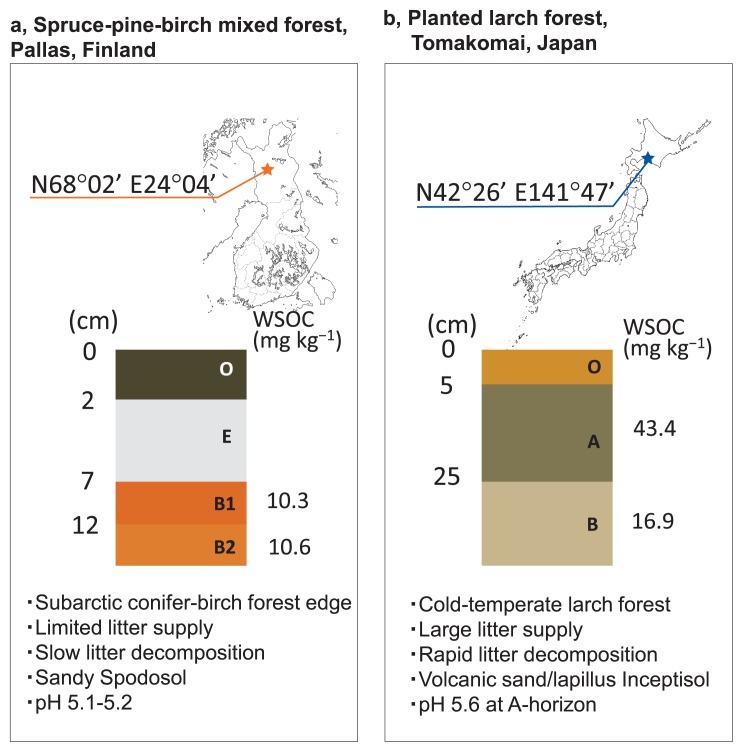
Location and soil profiles of two sampling sites in the conifer-birch forest edge, Finland, and cold-temperate volcanic lapilli, Japan. (a) The mixed conifer-birch forest edge, containing sparsely grown pine, spruce, and birch in a moderate slope with a smaller litter supply, opened toward a hilly heathland. (b) The planted larch forest supplied a large mass of leaf litter on the forest bed as a thick organic topsoil layer (O-horizon).

**Fig. 2:**
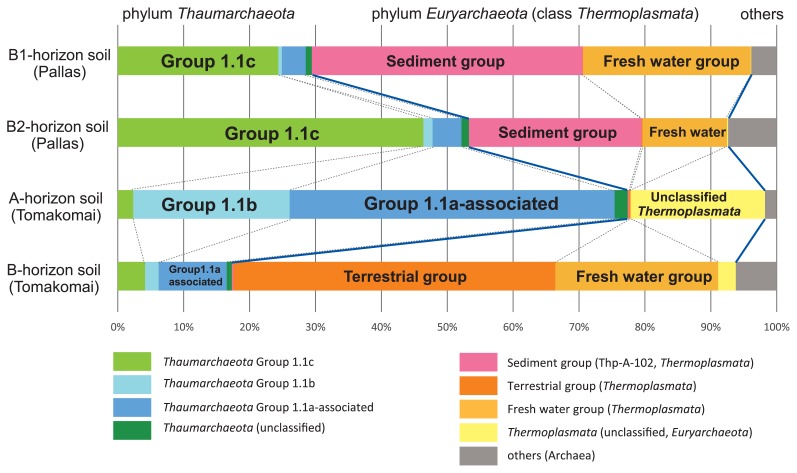
High-throughput analysis of archaeal 16S rRNA gene fragments from Pallas and Tomakomai mineral horizons of soils. The numbers of the mapped reads of the four samples used in this analysis (Pallas B1, Pallas B2, Tomakomai A, and Tomakomai B horizons) were 93732, 211468, 123628, and 209389, respectively. Sequences longer than 370 bp having a quality score of more than 20 without Ns were submitted to the RDP classifier using a bootstrap cut-off of 0.8. Blue solid lines show the boundary between the phylum *Thaumarchaeota* and class *Thermoplasmata* of the phylum *Euryarchaeota*, or between *Thermoplasmata* and other *Archaea*.

**Fig. 3:**
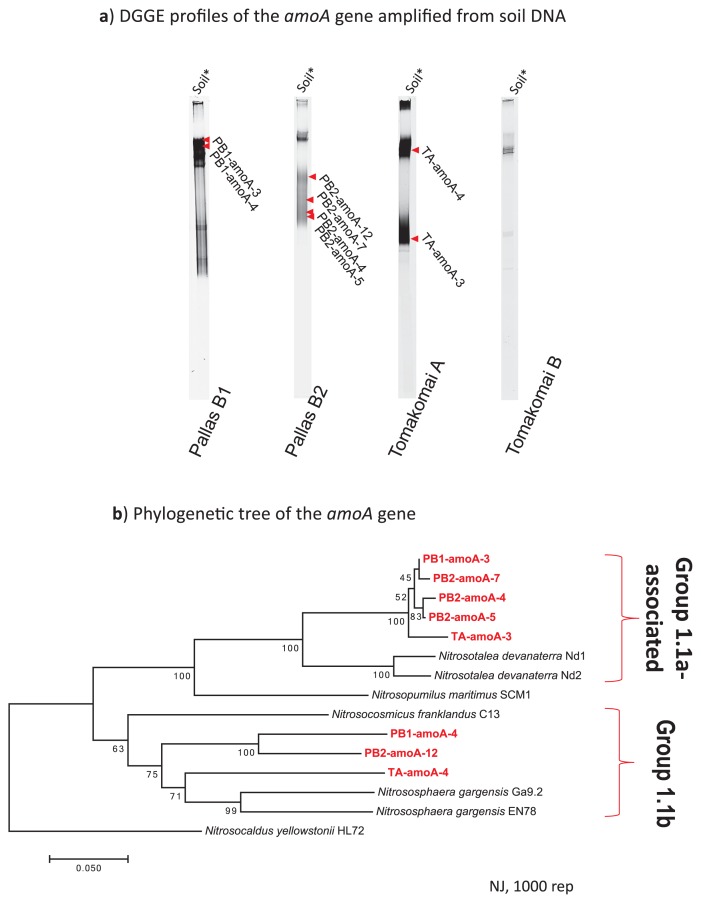
Denaturing gradient gel electrophoresis (DGGE) profile of the partial *amoA* gene obtained from soil samples, and phylogeny of *amoA* base sequences. a) DGGE profile of *amoA* for soil DNA extracted directly from each soil sample. Successfully sequenced DNA bands are shown with red arrows. b) Phylogenetic tree with some reference strains. Regarding clustering, we used the neighbor-joining method with 1000 bootstrap replicates. Clones of partial *amoA* genes (593 bp) are shown by red letters.
