# Supplementary material for: Comparison of Archaeal Communities in Mineral Soils at a Boreal Forest in Finland and a Cold-Temperate Forest in Japan
Source: Microbes Environ. 2017 Nov 7;32(4):390–3. doi: 10.1264/jsme2.ME17100 (PMC5745025; doi:10.1264/jsme2.ME17100)
Supplement: Supplementary file 1 [file 32_390_s1.pdf]

Supplemental information

Materials and methods

Soil sampling

In a mixed pine-spruce forest (estimated age of trees > 100 years) in Pallas National Park (68°02′N, 24°04′E, altitude 710 m), Finland, inside the Arctic Circle, soils in the B-horizons are poor in organic substances, and the soil archaeal communities were investigated. The soil samples were collected in early September 2009 from the forest edge. The sandy subsoil (B1- and B2-horizons) were sampled in triplicate, but we were unable to collect sufficient samples from relatively thin O-horizon soils (Fig. 1a). The soils were immediately kept at 4°C and transported to our laboratory at Hokkaido University. For comparison, archaeal communities in acidic volcanic lapilli soil (A-horizon) and lapilli subsoil (B-horizon) from a planted larch forest (> 40 years old) established on a volcanic sand/lapilli bed inside Tsutamori Forest Park in Tomakomai, Japan (42°39′N, 141°47′E, altitude 25 m) in late May, 2011 were also investigated (Fig. 1b).

For determination of soil pH, 3 g of each soil sample was mixed with 15 mL of deionized water and shaken for 30 min by hand, and the pH of the resulting soil suspension was measured using a Navi-H pH F-52 portable pH meter (Horiba, Kyoto, Japan). To determine the level of water-soluble organic carbon (WSOC), 2 g of each soil sample was mixed with 20 mL of MilliQ water and shaken for 1 h at 130 rpm. The aqueous extracts were then filtered through a 0.45 µm membrane and the organic carbon contained in the filtrate was measured using a TOC-5000A total organic carbon analyzer (Shimadzu, Kyoto, Japan).

DNA extraction

DNA was extracted from the soil samples using an ISOIL® Large for Beads ver.2 DNA extraction kit for soil (Nippon Gene, Toyama, Japan) with scaled down of the protocol 10-fold. A 0.5 g portion of each soil sample was subjected to the extraction process as described in the instruction manual, and the resulting DNA samples were quantified at wavelengths of 280- and 260-nm using a Gene Quant *Pro* RNA/DNA Calculator (Pharmacia, Uppsala, Sweden).

High-throughput sequencing

V4-5 region of archaeal 16S rRNA was amplified using nested PCR: 1st PCR with a primer pair of *arch23f* (5'-TGC GAY CTG GTY GAT YCT GCC-3') (Burggraf et al., 1991) and *arch1492r* (5'-TAC GGY TAC CTT GTT ACG ACT T-3') (Lane, 1991) followed by 2nd PCR with *arch519f* (5'-CAG CAG CCG CGG TAA TAC-3') (Øvreås et al., 1997) and *arch915R* (5'-GTG CTC CCC CGC CAA TTC CT-3') (Coolen *et al*., 2004). The cycling condition for 1st PCR was as follows: 95°C for 10 min, 30 cycles of 95°C for 30 sec, 60°C for 30 sec, 72°C for 1.5 min, then one cycle of 72°C for 7 min. The resulting amplicons (1.5 kbp) purified were subjected to 2nd PCR under the cycling condition as follows: 95°C for 10 min, 30 cycles of 95°C for 30 sec, 60°C for 30 sec, 72°C for 30 sec, then one cycle of 72°C for 7 min. The resulting amplicon with *519F/915R* was purified by Agencourt AMPure XP (Beckman Coulter). All the samples were quantified using a Qubit dsDNA HS assay kit (Life Technologies). The archaeal 16S rRNA gene libraries were prepared using the Ion Plus Fragment Library Kit (Life Technologies) and barcoded with the IonXpress Barcode Adapters 1-16 Kit (Life Technologies) to distinguish four soil samples. The library and template were prepared according to the manufacturer's instructions. The libraries were qualified and quantified using an Agilent 2100 Bioanalyzer with High Sensitivity DNA Analysis Kits (Agilent Technologies, CA, USA). Each library was diluted to 26 pM and subjected to emulsion PCR to amplify sequencing templates onto Ion Sphere Particles (ISPs), using the Ion OneTouch 2 system (Life Technologies), and enriched with the Ion OneTouch ES (Life Technologies) using the Ion PGM Template OT2 400 Kit (Life Technologies). The enriched ISPs were subjected to single-end sequencing using the Ion Torrent PGM system (Life

Technologies) using the Ion PGM Sequencing 400 Kit (Life Technologies) and Ion 316 Chip v2 (Life Technologies) for 850 flows.

The number of assembled short reads for 16S rRNA gene-targeted DNA sequences obtained from B1- and B2-horizon soils of Pallas and A- and B-horizon soils of Tomakomai and screened for quality control were 1828, 4110, 3322, and 3727, respectively (Huse *et al*., 2007). The sequences aligned with SILVA archaeal reference v119, ranging from 370 to 430 bp in length, were merged into 2477 unique sequences in total. Sequences showing differences at four or less bases were defined as the same sequence (Pruesse *et al.,* 2007), and the reads were subjected to a chimeric check using the UCHIME algorithm (Edgar *et al*., 2011); the resulting 1,894 unique sequences were classified using the Bayesian method with the SILVA reference v128 (Schloss *et al.,* 2011).

amoA gene targeted DGGE

The ammonia monooxygenase subunit A (*amoA*) gene of the archaea from the soils was also cloned after separation of *amoA* gene-targeted PCR-DGGE bands using degenerate primers $\textit{CrenamoA23}_{gc}\textit{f}$ (5′-CGC CCG CCG CGC CCC GCG CCC GTC CCG CCG CCC CCG CCC GAT GGT CTG GCT WAG ACG-3′) and *CrenamoA616r* (5′-GCC ATC CAT CTG TAT GTC CA-3′) (Muyzer *et al*., 1993; Avrahami *et al*., 2003; Tourna *et al.*, 2008). The cycling condition was as follows: 95°C for 10 min, 30 cycles of 95°C for 30 sec, 55°C for 30 sec, 72°C for 30 sec, then one cycle of 72°C for 7 min.

The DGGE analyses for the *amoA* gene amplicon was performed in 30-50% denaturing gradient gel using a DCode system (Bio-Rad, Hercules, CA, USA) at 60°C, 100V for 16 h (Kowalchuk et al., 1997; Tzeneva et al., 2008). The gel plate completed electrophoresis was dyed with SYBR Green I for 30 min and scanned by an image analyzer Typhoon 9400 (GE healthcare, Little Chalfont, UK). Using the *amoA* gene sequence from *Nitrososphaera*

vienensis EN76 genomic DNA as a positional standard (Tourna et al., 2011), all the sequences were clustered using MEGA v7.0.16.

References

Avrahami, S., Liesack, W., and Conrad, R. 2003. Effects of temperature and fertilizer on activity and community structure of soil ammonia oxidizers. Environ Microbiol. 5:691-705

Burggraf, S., Stetter, K.O., Rouviere, P., and C.R. Woese. 1991. Methanopyrus kandleri: an archaeal methanogen unrelated to all other known methanogens. Sys. Appl. Microbiol. 14:346–351.

Coolen, M.J.L,, Hopmans, E.C.W., Rijpstra, I.C. et al. 2004. Evolution of the methane cycle in Ace Lake (Antarctica) during the Holocene: response of methanogens and methanotrophs to environmental change. Org. Geochem. 35:1151–1167.

Edgar, R.C., Haas, B.J., Clemente, J.C., Quince, C., Knight R. 2011. UCHIME improves sensitivity and speed of chimera detection. Bioinformatics 27: 2194–2200.

Huse, S.M., Huber, J.A., Morrison, H.G., Sogin, M.L., and Welch, D.M. 2007. Accuracy and quality of massively parallel DNA pyrosequencing. Genome Biol. 8:R143.

Kowalchuk, G., Stephen, J. R., De Boer, W., Prosser, J. I., Embley, T. M., and Woldendorp, J. W. 1997. Analysis of ammonia-oxidizing bacteria of the beta subdivision of the class Proteobacteria in coastal sand dunes by denaturing gradient gel electrophoresis and sequencing of PCR-amplified 16S ribosomal DNA fragments. Appl. Environ. Microbiol., 63(4), 1489–1497.

Lane, D. J. 1991. 16S/23S rRNA sequencing. In: Stackebrandt E, GoodfellowM (eds) Nucleic acid techniques in bacterial systematics. JohnWiley & Sons, New York, NY, pp. 115–175.

Muyzer, G., de Waal, E. C., and Uitierlinden, A. G. 1993. Profiling of complex microbial populations by denaturing gradient gel electrophoresis analysis of polymerase chain reaction-amplified genes coding for 16S rRNA. Appl. Environ. Microbiol., 59: 695–700.

Øvreås, L., Forney, L., and Daae, F.L. 1997. Distribution of bacterioplankton in meromictic Lake Saelenvannet, as determined by denaturing gradient gel electrophoresis of PCR-amplified gene fragments coding for 16S rRNA. Appl. Environ. Microbiol. 63:3367–3373.

Pruesse, E., Quast, C., Knittel, et al. 2007. SILVA: a comprehensive online resource for quality checked and aligned ribosomal RNA sequence data compatible with ARB. Nucleic Acids Res. 35: 7188–7196.

Schloss, P.D., Gevers, D., and Westcott, S.L. 2011. Reducing the effects of PCR amplification and sequencing artifacts on 16S rRNA-based studies. PloS ONE 6:e27310.

Tourna, M., Freitag, T.E., Nicol, G.W., and Prosser, J.I. 2008. Growth, activity and temperature responses of ammonia-oxidizing archaea and bacteria in soil microcosms. Environ. Microbiol. 10: 1357–1364.

Tzeneva, V.A., Heilig, H.G., van Vliet, W.A., Akkermans, A.D., de Vos, W.M., & Smidt, H. 2008. 16S rRNA targeted DGGE fingerprinting of microbial communities. Methods Mol Biol., 410, 335–350.
